# Case report: Novel germline c.587delA pathogenic variant in familial multiple endocrine neoplasia type 1

**DOI:** 10.3389/fendo.2024.1467882

**Published:** 2024-09-20

**Authors:** Haotian Huang, Jianwei Li, Kun Zhang, Yu Tang, Min Zhang, Zhen Fan, Tao Wang, Yaoxia Liu

**Affiliations:** ^1^ School of Medicine, University of Electronic Science and Technology of China, Chengdu, China; ^2^ Department of Endocrinology and Metabolism, West China Hospital, Sichuan University, Chengdu, China; ^3^ School of Biological Sciences and Technology, Chengdu Medical College, Chengdu, China; ^4^ Department of Geriatric Endocrinology, Sichuan Provincial People’s Hospital, School of Medicine, University of Electronic Science and Technology of China, Chengdu, China; ^5^ Department of Pediatrics, West China Second University Hospital, Sichuan University, Chengdu, China; ^6^ Key Laboratory of Birth Defects and Related Diseases of Women and Children (Sichuan University), Ministry of Education, Chengdu, China

**Keywords:** multiple endocrine neoplasia type 1, MEN1 gene, menin, pathogenic variant, pediatric endocrinology

## Abstract

Multiple Endocrine Neoplasia type 1 (*MEN1*) is a rare genetic disease, characterized by co-occurrence of several lesions of the endocrine system. In *MEN1*, the pathogenic *MEN1* gene mutations lead to the Abnormal expression of menin, a critical tumor suppressor protein. We here reported a case of a 14‐year‐old male with insulinoma and primary hyperparathyroidism. Genetic testing demonstrated a novel heterozygote variant c.587delA of MEN1, resulting in the substitution of the 196th amino acid, changing from glutamic acid to glycine, followed by a frameshift translation of 33 amino acids. An identical variant was identified in the proband’s father, who was further diagnosed with hyperparathyroidism. To the best of our knowledge, this is the first report of MEN1 syndrome caused by the c.587delA MEN1 variant. Observations indicated that, despite sharing the same MEN1 gene change, family members exhibited diverse clinical phenotypes. This underscored the presence of genetic anticipation within the familial context.

## 1 Introduction

Multiple Endocrine Neoplasia type 1 (MEN1) is a rare genetic disease, characterized by co-occurrence of several lesions of the endocrine system (1, 2). Prevalence of MEN1 is 3–10/100,000 (1). MEN1 most frequently involves the parathyroids and pancreas, pituitary gland, adrenal glands, lungs, and thymus in descending order of occurrence. The extent of penetrance also depends on the specific endocrine organ affected. The disease’s penetrance increases with age, beginning as early as 10 years old and reaching near-universal levels by 60 years of age (3). Transmission is autosomal dominant, linked to heterozygous inactivating variants of the MEN1 gene, located at 11q13 (1, 4). Spanning 9 kilobases with 10 exons, the MEN1 gene encodes the protein menin, which consists of 610 amino acids and plays a crucial role in various cellular mechanisms through interactions with numerous partners (1, 5). Functioning as a tumor suppressor gene, MEN1 gene changes result in neuroendocrine tumors (NETs) via the “double hit” hypothesis proposed by Knudson (1).

According to the diagnostic criteria established in 2012 (2), MEN1 can be diagnosed by one of three criteria: on clinical criteria, by presence of at least 2 major MEN1 lesions; on familial criteria, by presence of a MEN1 lesion in a first-degree relative of an individual presenting clinical MEN1; on genetic criteria, in an individual presenting a pathogenic or probably pathogenic variant of the MEN1 gene, whether symptomatic or not. Patients presenting with clinical MEN1 or suspected MEN1 according to the criteria should undergo genetic analysis to screen for MEN1 gene abnormality. Any pathogenic or probably pathogenic variant of the MEN1 gene thus identified must be checked on a second sample (1). Family members of patients clinically diagnosed with MEN1 who develop a MEN1-associated tumor meet the criteria for a familial MEN1 diagnosis (2).

This case report describes a family with MEN1 due to a novel heterozygous pathogenic variant,c.587delA. This variant leads to the substitution of the 196th amino acid, changing from glutamic acid to glycine, followed by a frameshift that results in the translation of an additional 33 amino acids before premature termination. Moreover, this case highlights the variability in disease phenotypes among family members carrying the same gene change.

## 2 Case presentation

**Figure 1:**
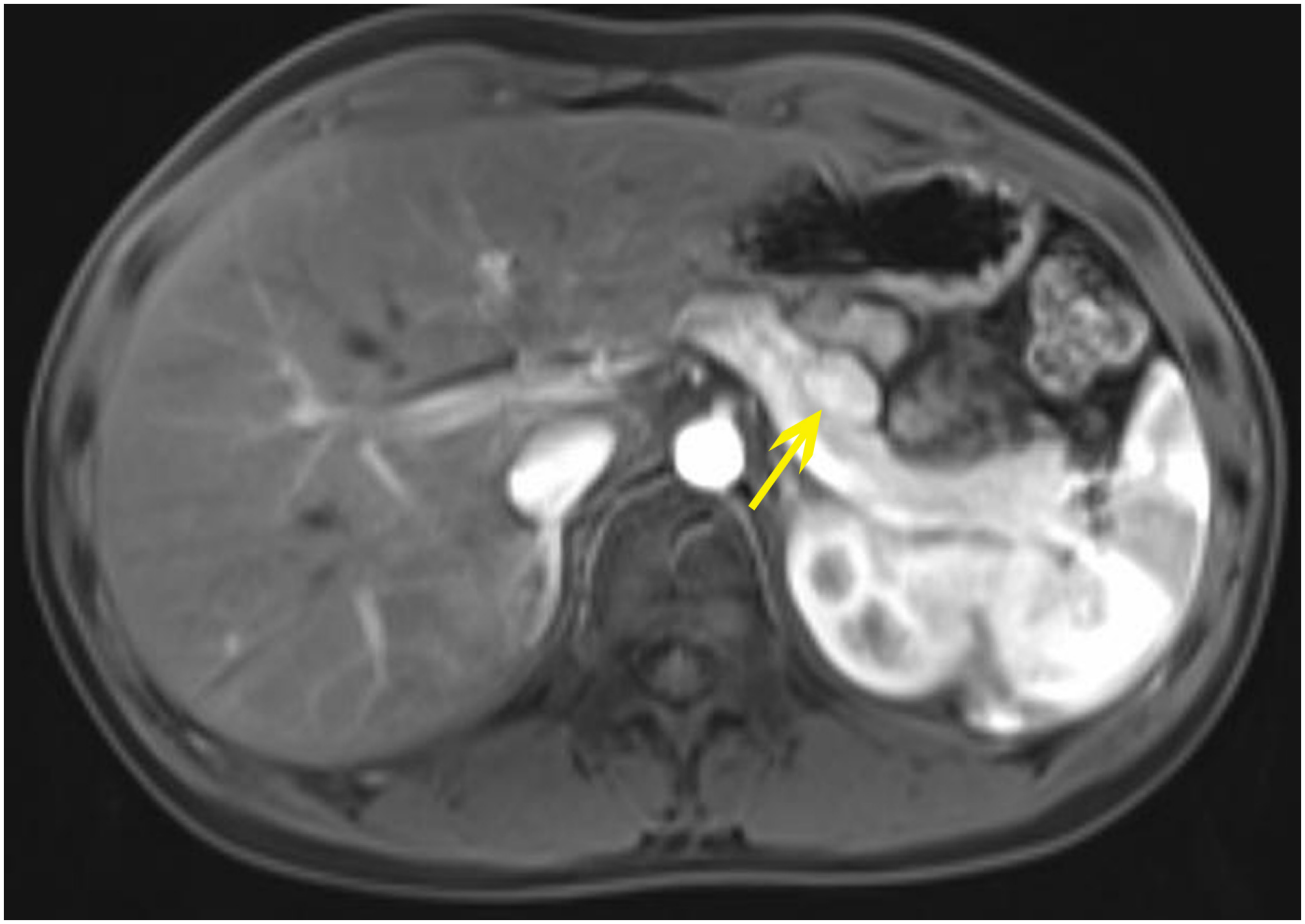
The abdominal MRI with contrast of the proband shows: a nodular lesion approximately 1.7x1.2 cm in size located in the anterior part of the pancreatic body, displaying slightly prolonged T1 and T2 signals. Diffusion is suspiciously restricted, with marked enhancement post-contrast, consistent with the enhancement of the pancreatic parenchyma.

The index case, a 14-year-old male, presented with recurrent seizure-like episodes for over 2 years. Subsequent to an episode of unrousability upon awakening, fasting blood glucose was found to be 2.09 mmol/L (normal range 3.9-5.9), insulin levels at 23.96 μU/ml (normal range 1.5-15), and C-peptide at 0.972 nmol/L (normal range 0.48-0.78). Enhanced abdominal Magnetic Resonance Imaging(MRI) scanning indicated a nodular lesion in the anterior part of the pancreatic body, suggestive of a neuroendocrine tumor(**Figure 1**). Physical examination upon admission did not reveal any abnormalities.

## 3 Diagnostic assessment

On January 29, 2019, the patient underwent distal pancreatectomy and partial pancreatectomy under general anesthesia. Pancreatic pathology reported tumor cells positive for PCK (dot-like+), CgA (+), Syn (+), CD56 (+), ATRX (+), Rb (sporadically+), P53 (+, 5%), with a Ki-67 positivity rate of 5%-10%, diagnosing it as a Grade 2 neuroendocrine tumor (NET G2). Immunohistochemistry showed positivity for insulin; glucagon, gastrin, and somatostatin were negative. The final diagnosis was an insulinoma. Post-surgery, fasting blood glucose levels ranged from 4.28 to 5.02 mmol/L, and fasting insulin levels varied from 6.28 to 13.18 μU/ml, with no post-operative seizure-like episodes.

**Figure 2:**
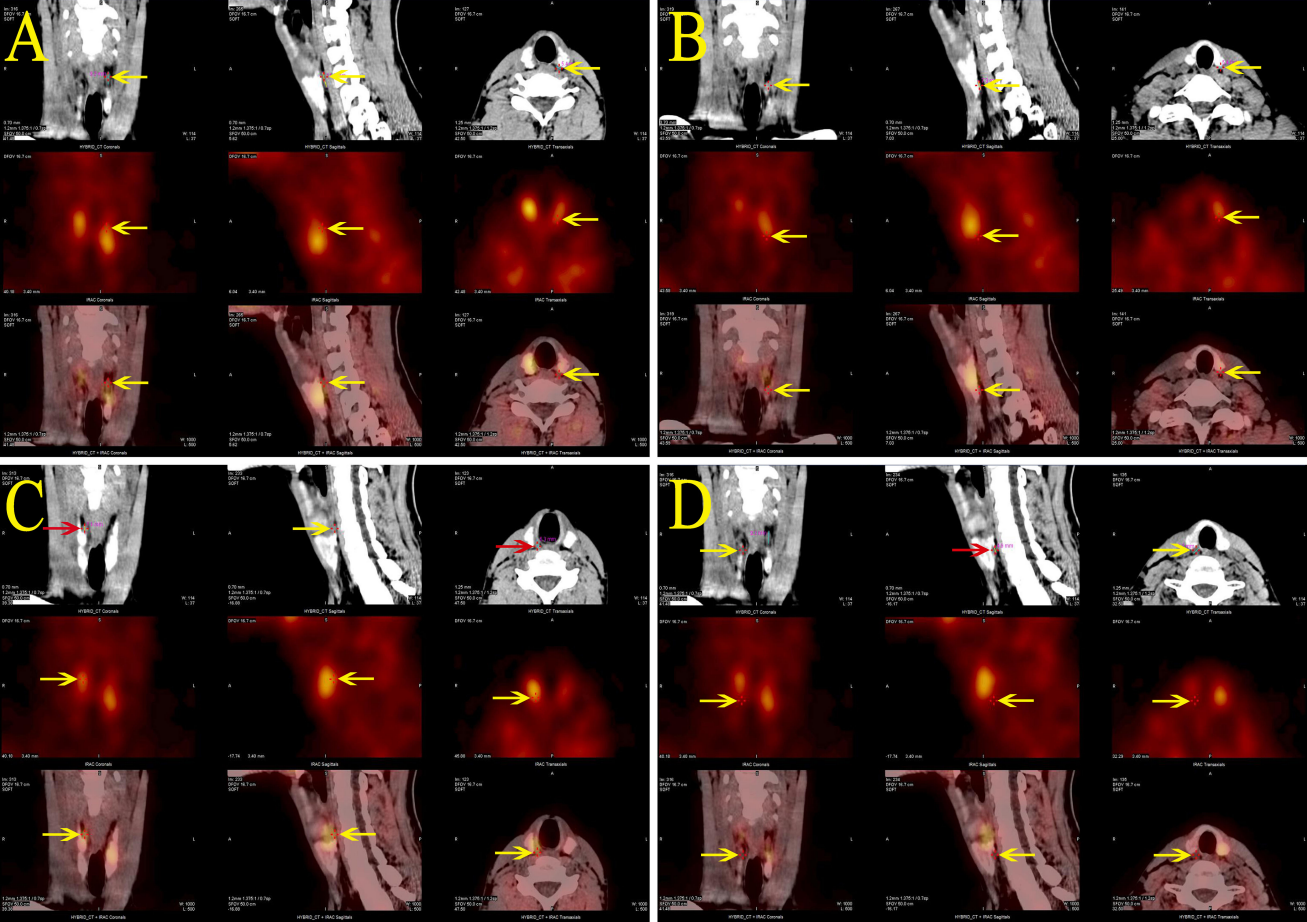
Parathyroid SPECT/CT hybrid imaging of the proband: four areas of low-density shadows located posteriorly in the upper portions of both thyroid lobes **(A, C)**, the posterior inferior part of the left lobe **(B)**, and the posterior middle part of the right lobe **(D)**, suggesting a parathyroid tissue origin. However, none of these areas demonstrated significant MIBI uptake.

Calcium, phosphate, and PTH levels were normal during the hospital stay. But during follow-up, elevated blood calcium, PTH levels, and decreased phosphate levels were observed. Blood calcium levels were monitored at 2.62-2.81 mmol/L (normal range 2.1-2.7 mmol/L), phosphate at 1.07-1.33 mmol/L (normal range 0.81-1.45 mmol/L), and PTH at 9.85-11.35 pmol/L (normal range 1.60-6.90 pmol/L), with a 24-hour urinary calcium excretion of 12.94 mmol/24h (normal range 2.5-7.5) and urinary phosphate of 27.0 mmol/24h (normal range 22-48). Bone metabolism markers indicated a significant increase in bone turnover. The patient experienced no symptoms of polydipsia, polyuria, nausea, vomiting, abdominal pain, back pain, hematuria, or bone pain. Parathyroid ultrasound revealed: bilateral deep parathyroid solid nodules. Parathyroid single photon emission computed tomography/computed tomography (SPECT/CT) hybrid Imaging: four parathyroid glands were visualized, and there was no significant increase in parathyroid technetium-99m methoxyisobutylisonitrile (99mTc-MIBI) scan uptake (**Figure 2**).

Bone density was within the normal range for the same age group. Urinary system ultrasound showed no urinary tract stones. On July 3, 2019, under general anesthesia, “neck exploration + total parathyroidectomy + partial autotransplantation of the parathyroid gland” was performed. Paraffin pathology diagnosis of the upper left, lower left, and upper right parathyroid tissues indicated hyperplasia. The surgery was successful, with preoperative PTH measured at 11.44 pmol/L and postoperative PTH at 30 min measured at 1.15 pmol/L. Postoperative treatment included calcium supplementation and vitamin D. Four weeks postoperatively, PTH was 1.98 pmol/L, blood calcium 2.33 mmol/L, and phosphate 1.7 mmol/L. Medications were gradually discontinued, and follow-up to date shows normal blood calcium, phosphate, PTH levels, with no hypoglycemia or seizure episodes, and no symptoms or biochemical markers of other MEN1-associated systemic diseases.

Given the patient’s concurrent presentation with an insulinoma and hyperparathyroidism, we conducted screening for MEN1-associated disease phenotypes and genetic testing. The patient’s gastrin levels, pituitary and target gland axis hormones, blood and urine catecholamines and their metabolites, aldosterone-renin-angiotensin system, complete blood count, liver and kidney function tests, urinalysis, and tumor markers (AFP, CEA, CA19-9, CYFRA21-1, NSE) were all within normal ranges. Ultrasounds of the kidneys, adrenal glands, and scrotum, as well as chest and head CT scans, showed no abnormalities. Pituitary MRI revealed no mass lesions.

**Figure 3:**
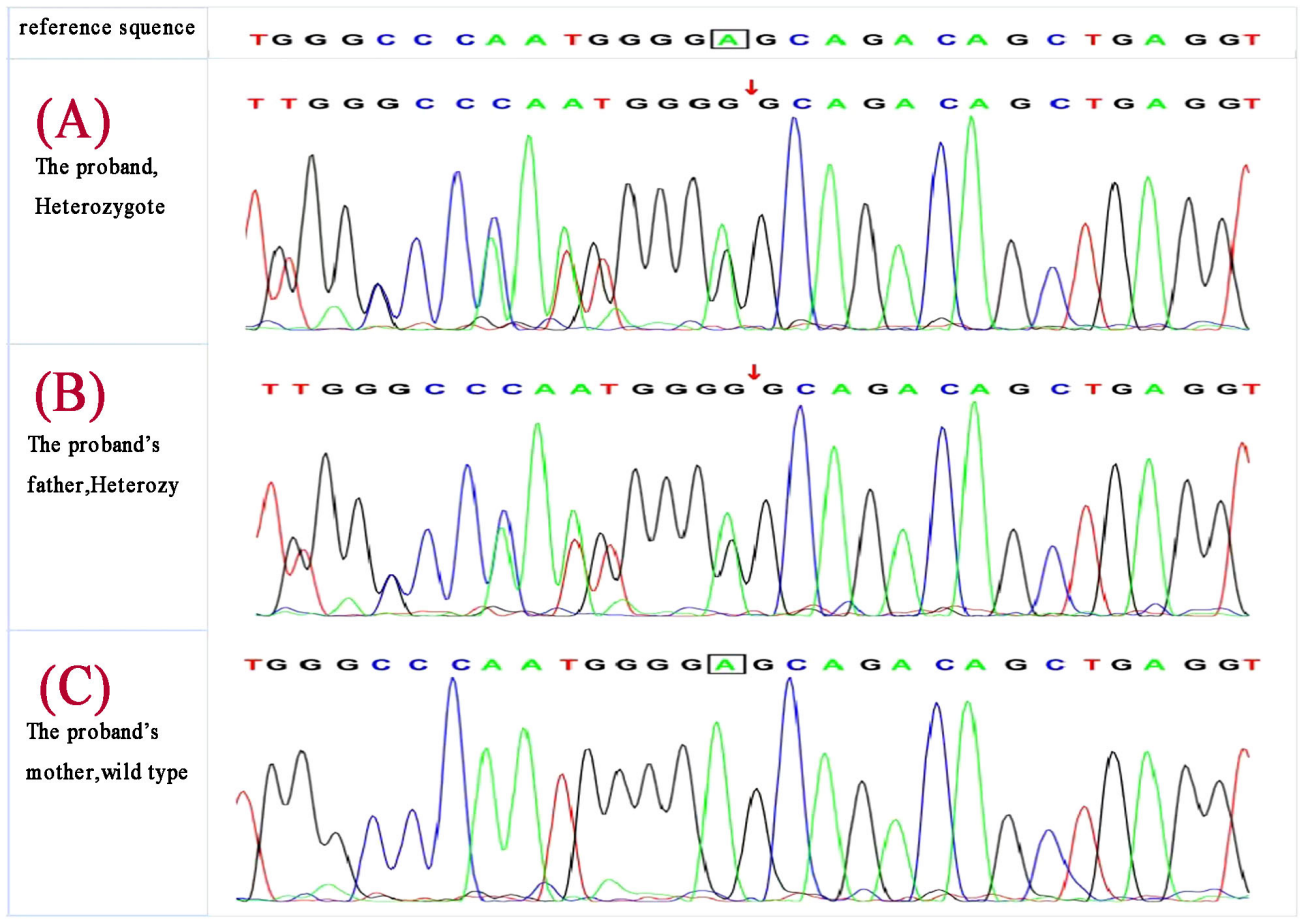
Deepened whole-exome sequencing identified a genetic variant c.587delA on chromosome 11 (chr11:64575445) in the proband **(A)**, the proband’s father carried the same gene change **(B)** ,and the mother was wild type **(C)**.

Deepened whole-exome sequencing of the patient identified a genetic variant c.587delA on chromosome 11 (chr11:64575445) (**Figure 3A**), resulting in an amino acid sequence change of p.Glu196GlyfsTer33(NM_130803). This denotes a base A deletion at position 587 in the coding region, leading to the substitution of the 196th amino acid from glutamic acid to glycine, followed by a frameshift translation of 33 amino acids before translation termination. The total sequencing depth was 144, with a variant depth of 84, indicating heterozygosity. According to the 2015 ACMG guidelines, the variant is classified as likely pathogenic: PVS1 (loss-of-function variant likely results in gene inactivation) + PM2 (MAF <0.005, considered a low-frequency variant).

**Figure 4:**
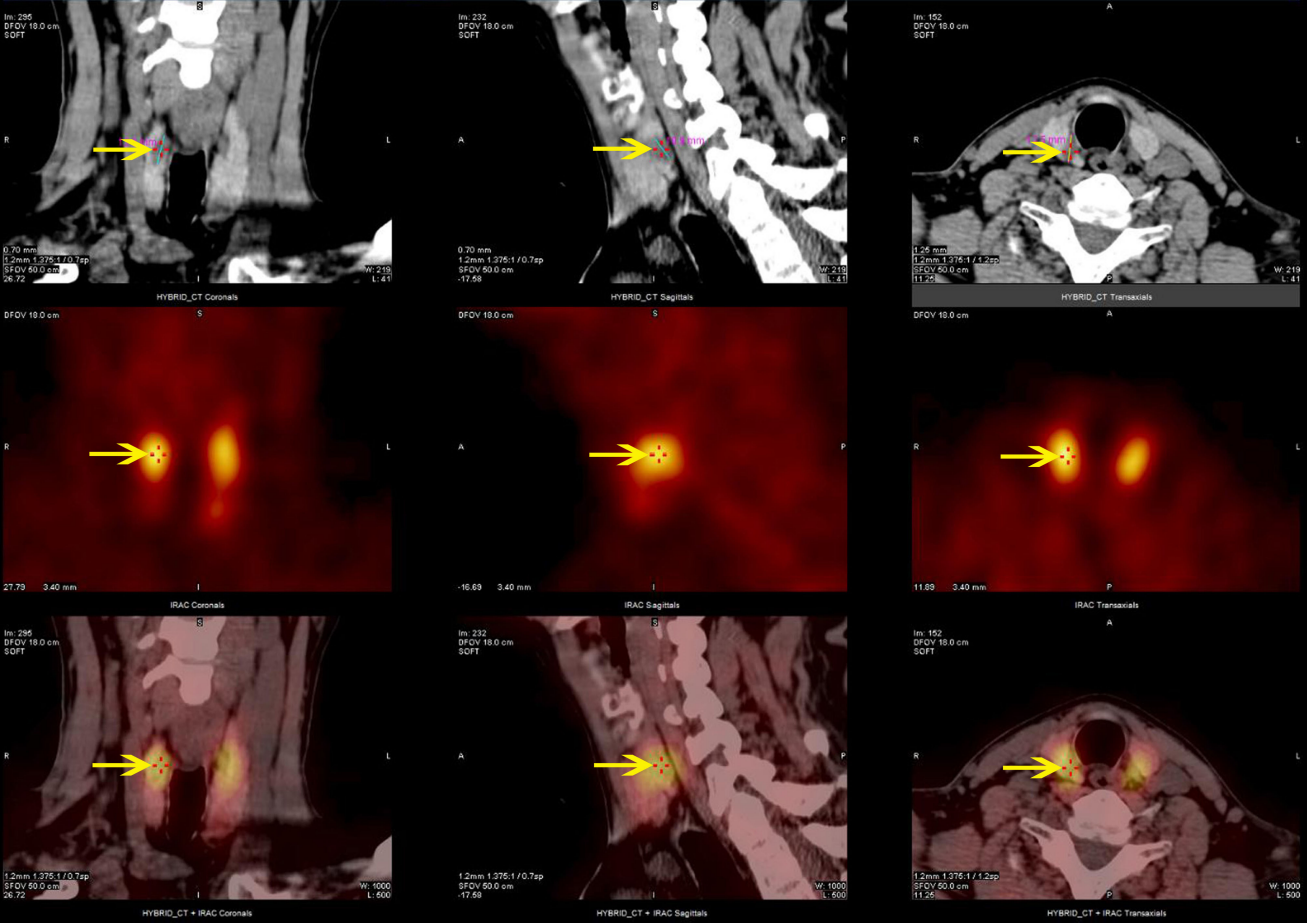
Parathyroid SPECT/CT hybrid imaging of the proband’s father: nodular parathyroid imaging is observed in the posterior aspect of the lower pole of the right thyroid lobe, with mildly increased MIBI uptake.

MEN1 gene testing was performed on the index case’s parents. The father carried the same gene change, also as a heterozygote(**Figure 3B**), and the mother was wild type (**Figure 3C**). The father, a 43-year-old male with a history of recurrent back pain for over 6 years, had undergone multiple procedures for bilateral ureteral stones, including extracorporeal shock wave lithotripsy and transurethral ureterolithotomy, with postoperative analysis confirming the stones as calcium oxalate. Physical examination revealed no positive findings. Further tests showed parathyroid hormone (PTH) at 24.28 pmol/L, blood calcium at 2.77 mmol/L, serum inorganic phosphorus at 0.67 mmol/L, and fasting blood glucose at 5.67 mmol/L. A 24-hour urine electrolyte analysis indicated calcium at 4.38 mmol/24h and phosphorus at 16.20 mmol/24h. Bone metabolism markers suggested a significant increase in bone turnover. Gastrin, pituitary and target gland axis hormones, blood and urine catecholamines and their metabolites, aldosterone-renin-angiotensin system, fasting blood glucose, insulin, C-peptide, complete blood count, liver and kidney function, urinalysis, and tumor markers were all normal. Enhanced MRI of the pancreas and pituitary MRI showed no definitive abnormalities. Parathyroid SPECT/CT hybrid imaging revealed mild increased MIBI uptake in a nodule behind the lower part of the right thyroid lobe, suggesting parathyroid tissue origin (**Figure 4**). On July 9, 2019, a neck exploration and resection of the right lower parathyroid adenoma were performed, in line with the patient’s preference against subtotal parathyroidectomy. Pathology indicated adenomatous hyperplasia of the right lower parathyroid, with immunohistochemistry showing PTH (+), CD56 (-), Syn (+, partial), CgA (+), and a Ki-67 positivity rate of approximately 1-2%, special staining for reticular fibers (Foot) was positive. Postoperative reevaluation showed blood calcium at 2.51 mmol/L and phosphate at 0.95 mmol/L. Follow-up to the present day, February 18, 2024, shows the father with normal blood calcium, phosphate, PTH levels, no further episodes of urinary tract stones, and no emergence of other MEN1-associated disease phenotypes.

The proband has no siblings, and his grandparents and father’s siblings were unwilling to undergo genetic testing, opting only for screening of blood glucose, calcium, phosphate, and PTH, all of which were normal. In this case, the c.587delA gene variant in the index case as a heterozygote shows co-segregation with the phenotype and genotype of the father, consistent with autosomal dominant inheritance. The c.587delA variant is confirmed as the pathogenic gene variant causing familial MEN1. In summary, we have compiled a comprehensive table detailing the therapeutic interventions and laboratory test results pertaining to the proband and his father throughout the course of their diagnosis and treatment. This information can be found in **Table 1** of the supplementary materials.

**Table 1:** Results of the proband’s and his father’s laboratory tests from diagnosis to four weeks postoperatively.

Parameter (reference range and unit)	At the time of Diagnosis (The proband, January, 2019)	Surgery (January 29, 2019)	Six-month postoperative follow-up	Surgery (July 3, 2019)	Four weeks postoperative	At the time of Diagnosis (the proband’s father, July, 2019)	Surgery (July 9,2019)	Four weeks postoperative
FBG (3.9-5.9mmol/L)	2.09	Distal pancreatectomy and partial pancreatectomy	4.28-5.02	neck exploration + total parathyroidectomy + partial autotransplantation of the parathyroid gland	NA	5.06	Neck exploration and resection of the right lower parathyroid adenoma	NA
INS (1.5-15μU/ml)	23.96		6.28-13.18		NA	18.80		NA
C-P (0.48-0.78 nmol/L)	0.972				NA	NA		NA
Ca (2.1-2.7mmol/L)	2.69		2.62-2.81		2.33	2.77		2.51
P (0.81-1.45mmol/L)	0.90		1.07-1.33		1.37	0.67		0.95
PTH (1.6-6.9pmol/L)	6.85		9.85-11.35		1.98	24.28		4.2

FBG, fasting blood glucose; INS, Insulin; C-P, C-peptide; Ca, calcium; NA, not available; P, phosphorus; PTH, parathyroid hormone.

### 3.1 Material and methods

Whole-exome sequencing was performed with the IDT xGen Exome Research Panel v1.0 for exome capture, followed by sequencing on the Illumina NovaSeq 6000 series sequencer. The sequencing coverage for targeted sequences was no less than 99%.

## 4 Discussion

The MEN1 gene encodes the menin protein, which is a scaffold protein that is involved in histone modification and epigenetic gene regulation (6), playing a role in tumor suppression associated with MEN1. Menin interacts with over 50 different known proteins, influencing various cellular processes like cell cycle progression, DNA repair, and transcriptional regulation. It interacts with transcription factors and chromatin-modifying proteins, affecting pathways like TGF-β/BMP, nuclear receptors, Wnt/β-catenin, and Hedgehog, crucial for gene expression regulation. Loss of menin affects these interactions, impeding these signaling pathways and their anti-proliferative effects (7).The inactivation of menin due to genetic variants can lead to the loss of its tumor-suppressive function. Pathogenic variants of MEN1 usually have a truncating effect on menin, with loss of the tumor-suppressing function and an increased risk of developing cancer (8, 9).

More than 1,300 variants in the MEN1 gene have been identified, distributed throughout the open reading frame, primarily in the coding exons as well as in intron sequences, without significant clustering or notable hot spots (9, 10). Approximately 69% of these germline variants in MEN1 are deemed pathogenic, leading to early truncation of menin. This is predominantly due to frame-shift variants (42%) and nonsense variants (14%), along with exon region deletions resulting from splicing defects (10.5%) and extensive deletions (2.5%) (8).

If a gene change that has not been previously reported is considered pathogenic, confirming its pathogenicity often requires demonstrating the same variant in another affected first-degree relative (11).The clinical diagnosis of MEN1 relies on detecting neoplastic disorders in at least two organs typically involved, such as the parathyroid glands, the anterior pituitary, and the pancreas. For our young male patient, pancreas was the initial sign of MEN1, followed by the discovery of Insulinoma and primary hyperparathyroidism. Genetic analysis identified a novel heterozygous c.587delA MEN1 variant, results in the substitution of the 196th amino acid from glutamic acid to glycine, followed by a frameshift translation of 33 amino acids before translation termination. Genetic validation demonstrated the same variant was also detected in his father, who was further diagnosed with hyperparathyroidism. In the case of the patient’s father, identical MEN1 symptoms as observed in his son were identified, allowing for the validation of the variant’s pathogenicity in a novel context. It was thought that such c.587delA MEN1 variant and frame-shift variants resulted in aberrant translation of menin, thereby impairing its tumor-suppressing function, and then leading to the occurrence of MEN1.

Observations indicated that, despite sharing the same MEN1 gene change, the family members exhibited diverse clinical phenotypes. The father developed the condition only in adulthood, exhibiting Solitary parathyroid adenoma, while the son experienced severe hypoglycemia symptoms due to an insulinoma before the age of 12, and subsequently developed hypercalcemia caused by hyperplasia of multiple parathyroid glands. This underscored the presence of genetic anticipation within the familial context. Considerable variability in the age of onset, clinical symptoms, disease severity, and types of tumors has previously been documented (12). The presentation of affected glands and their specific pathologies, such as hyperplasia or solitary or multiple parathyroid adenomas, can vary among family members, including identical twins (2, 13). Numerous efforts to analyze clinical characteristics in patients and their relatives with identical variants have verified the absence of straightforward correlations between phenotype and genotype (9, 12, 14, 15). It has been proposed that epigenetic mechanisms activated by environmental factors may affect the phenotype in individuals carrying the identical MEN1 variant (16).The clinical phenotype is therefore heterogeneous both for the variable penetrance of the disease and for the possible influences due to gene-environment interactions (17).

In our case study, whole-exome sequencing was performed on the patient and his parents. But, unfortunately, other family members of the patient were unwilling to undergo genetic testing, and we were unable to construct a detailed pedigree of the disease. We intend to maintain ongoing surveillance of the patient, his offspring, and their family relatives to observe subsequent changes in the MEN1 disease phenotype.

## 5 Conclusions

To our best understanding, this represented a familial MEN1 attributable to the novel MEN1 variant, c.587delA., which resulted in frameshift translation and abnormal expression of menin. Both the proband and his father were carriers of this identical variant, while exhibited diverse clinical phenotypes and varied symptom severity. Genetic testing is crucial for patients with MEN1 and epigenetic mechanisms may affect the phenotype in individuals carrying the identical variant.

## Data Availability

The original contributions presented in the study are publicly available. This data can be found here: https://doi.org/10.6084/m9.figshare.27021544.v1.
